# Altered sleep composition after traumatic brain injury does not affect declarative sleep-dependent memory consolidation

**DOI:** 10.3389/fnhum.2015.00328

**Published:** 2015-06-05

**Authors:** Janna Mantua, Keenan M. Mahan, Owen S. Henry, Rebecca M. C. Spencer

**Affiliations:** ^1^Neuroscience and Behavior Program, University of MassachusettsAmherst, MA, USA; ^2^Commonwealth Honors College, University of MassachusettsAmherst, MA, USA; ^3^Department of Psychological and Brain Sciences, University of MassachusettsAmherst, MA, USA

**Keywords:** sleep, memory consolidation, TBI, traumatic brain injury, NREM

## Abstract

Individuals with a history of traumatic brain injury (TBI) often report sleep disturbances, which may be caused by changes in sleep architecture or reduced sleep quality (greater time awake after sleep onset, poorer sleep efficiency, and sleep stage proportion alterations). Sleep is beneficial for memory formation, and herein we examine whether altered sleep physiology following TBI has deleterious effects on sleep-dependent declarative memory consolidation. Participants learned a list of word pairs in the morning or evening, and recall was assessed 12-h later, following an interval awake or with overnight sleep. Young adult participants (18–22 years) were assigned to one of four experimental groups: TBI Sleep (*n* = 14), TBI Wake (*n* = 12), non-TBI Sleep (*n* = 15), non-TBI Wake (*n* = 15). Each TBI participant was >1 year post-injury. Sleep physiology was measured with polysomnography. Memory consolidation was assessed by comparing change in word-pair recall over 12-h intersession intervals. The TBI group spent a significantly greater proportion of the night in SWS than the non-TBI group at the expense of NREM1. The TBI group also had marginally lower EEG delta power during SWS in the central region. Intersession changes in recall were greater for intervals with sleep than without sleep in both groups. However, despite abnormal sleep stage proportions for individuals with a TBI history, there was no difference in the intersession change in recall following sleep for the TBI and non-TBI groups. In both Sleep groups combined, there was a positive correlation between Intersession Change and the proportion of the night in NREM2 + SWS. Overall, sleep composition is altered following TBI but such deficits do not yield insufficiencies in sleep-dependent memory consolidation.

## Introduction

Over 1.7 million incidences of traumatic brain injury (TBI), ranging from mild concussions to severe head trauma, are recorded each year through emergency department visits, hospitalizations, and deaths (Coronado et al., 2011). Notably, even a single, mild TBI may have long-term psychological and physiological consequences [Armed Forces Health Surveillance Center (AFHSC), 2013; Bhalerao et al., 2013], as TBI encompasses both a primary mechanical insult and also a prolonged secondary injury. Secondary injury may be sustained through lactic acid accumulation from local glycolysis, edema, breakdown of the blood brain barrier, excessive neurotransmitter release, lipase activity, protease activity, and apoptosis (Werner and Engelhard, 2007). This assortment of physiological and chemical abnormalities may create adverse, long-lasting symptoms, including short-term memory deficits (Dean and Sterr, 2013) and an increase in subjectively disturbed sleep [Pillar et al., 2003; Verma et al., 2007; Orff et al., 2009; Armed Forces Health Surveillance Center (AFHSC), 2013; Bhalerao et al., 2013].

Subjective sleep complaints are likely rooted in physiological changes in sleep. Objective measures of sleep reveal decreased sleep efficiency (time asleep/time in bed) and increased wake after sleep onset time (WASO) following TBI (Kaufman et al., 2001; Parcell et al., 2008; Shekleton et al., 2010). An increased need for sleep, evidenced by self-reported and objectively measured hypersomnia (Baumann et al., 2007; Kempf et al., 2010; Sommerauer et al., 2013; Imbach et al., 2015) has also been found in post-TBI individuals. Additionally, overnight sleep recordings after chronic TBI show alterations in sleep architecture. Reports of sleep architecture vary, however, suggesting either lower percentage of sleep in non-rapid eye movement stage 2 (NREM2) and REM (Parcell et al., 2008; Schreiber et al., 2008) or a higher percentage of Slow Wave Sleep (SWS; Parcell et al., 2008; Shekleton et al., 2010; Sommerauer et al., 2013) when compared to those without a history of TBI. Finally, alterations in spectral power, a measure of synaptic strength and synchronization, have also been found following injury (Khoury et al., 2013).

Atypical sleep architecture may exacerbate long-term cognitive deficits caused by injury, as sleep is beneficial for the formation of new memories. Specifically, sleep rich in NREM2 and SWS facilitates declarative memory consolidation, which is the stabilization and transfer of memory traces from short-term to long-term memory (Plihal and Born, 1997; Stickgold, 2005; Diekelmann and Born, 2010; Wilson et al., 2012). For instance, it has been suggested that, during NREM2, declarative memory traces are reactivated and redistributed from the hippocampus to neocortex (Kempf et al., 2010), and this transfer is enhanced through increased cortical synchronization that occurs during SWS (Sirota et al., 2003; Takashima et al., 2006). Moreover, specific traits of NREM2 (e.g., sleep spindles or sigma power; Clemens et al., 2005; Genzel et al., 2009; Holz et al., 2012; Ruch et al., 2012) and SWS (e.g., %SWS and delta power; Holz et al., 2012) have been linked with declarative memory performance following a sleep bout (Marshall et al., 2006), particularly early in the night (Plihal and Born, 1997; Genzel et al., 2009). Combined NREM measures (e.g., spindle density across both stages and total %NREM; Plihal and Born, 1997; Clemens et al., 2005; Holz et al., 2012) have also been linked with overnight consolidation. The latter studies align with recent notions that sleep stages may act sequentially or in concordance to have beneficial declarative memory consolidation effects (Mazzoni et al., 1999; Ficca et al., 2000; Clemens et al., 2005; Göder et al., 2007; Ruch et al., 2012).

Despite well-established sleep deficits following brain injury, to our knowledge, sleep-dependent memory consolidation has never been examined in individuals with a history of TBI. Given that both NREM2 and SWS stages have reportedly been affected by TBI, we sought to examine whether these changes in sleep impair sleep-dependent consolidation. To this end, we utilized a word-pair learning task and investigated memory following intervals containing overnight sleep or spent fully awake. We used polysomnography, a system that objectively quantifies sleep quality and architecture, to examine sleep physiology in those with a history of mild TBI (i.e., having suffered from a concussion) compared to controls without a history of TBI. We hypothesized that TBI subjects would have impaired sleep, in measures of sleep stage duration and fragmentation (i.e., lower sleep efficiency and greater WASO) compared to those without a history of TBI. Moreover, we posited that poorer sleep quality would hinder sleep-dependent memory consolidation when compared to those without a history of TBI.

## Methods

### Participants

Participants were 58 healthy young adult participants (18–22 years). Subjects were recruited through an online recruitment system (SONA) or by word-of-mouth and were compensated with extra credit for a Psychology course or monetary payment. To be eligible, participants were required to habitually sleep more than 6 h per night and have less than 3 naps per week. Participants were excluded if they typically consumed more than 14 cups of coffee or alcohol per week, had a neurological disorder (other than a history of TBI in the TBI group), were taking anti-psychotic or anti-seizure medications, or were taking sleep-affecting medications. Importantly, to specifically examine the chronic effects of TBI (as opposed to transient, acute effects), participants in the TBI group had a history of TBI with injury at least 1 year prior to participation in the study.

Participants were assigned to one of four groups: TBI Wake, TBI Sleep, non-TBI Wake, or non-TBI Sleep. Participants were first assigned to a group based on TBI history and then semi-randomly assigned to a Sleep or Wake group, with careful balance of numbers of TBI incidents and severity of TBIs across the TBI Sleep and TBI Wake groups.

### Word-Pair Learning Task

The word-pair task has been utilized to examine declarative memory consolidation in previous studies in our lab (Wilson et al., 2012). Stimuli were 40 semantically-unrelated word pairs consisting of single-syllable nouns. The task had three phases: Encoding, Immediate Recall, and Delayed Recall. During Encoding, word pairs appeared on a computer monitor for 5 s with a 100 ms inter-stimuli interval. Participants passively viewed the pairs and were instructed to use a mnemonic strategy to help remember the pairs. Specifically, participants were told to think of associations between the pairs and to picture this association in their mind (e.g., if “aunt-zoo” was presented, they could imagine their aunt on display at a zoo). This instruction was designed to facilitate hippocampal-dependent contextual learning (Toki et al., 2014). Subsequently, participants practiced recalling the pairs with feedback. The first word from each pair was presented individually in a random order. The first and last two pairs of the encoded list were removed to eliminate primacy and recency effects (leaving a total of 36 pairs). If the participant’s response was incorrect, the correct response was displayed on the computer monitor for 750 ms. If the response was correct, the next stimuli appeared on the screen. Recall practice continued until participants reached 65% proficiency or the full list of words had appeared five times.

The Immediate Recall phase began following the Encoding phase. Participants were presented with one word from the encoded pairs and were instructed to recall the corresponding word in the pair. There was no feedback for correct or incorrect responses. In Session 2, which occurred after a 12-h break, participants completed the Delayed Recall phase, which was identical to the Immediate Recall phase.

### Procedure

Procedures were approved by the Institutional Review Board at the University of Massachusetts, Amherst. Before the study commenced, each participant provided written informed consent. Participants then underwent one of two in-lab sessions. For those in the Wake group, session 1 took place in the morning and session 2 took place in the evening, following 12 h awake. For the Sleep group, session 1 took place in the evening and session 2 took place the next morning, following 12 h containing an overnight sleep interval.

In the first session, after completing the consent process and the Stanford Sleepiness Scale (SSS), Morningness-Eveningness Questionnaire (MEQ), Epworth Sleepiness Scale (ESS), Pittsburgh Sleep Quality Index (PSQI), forward digit span test, and TBI questionnaire (all described below), participants performed the Encoding and Immediate Recall phases. Participants returned 12 h later to complete the Delayed Recall phase. Subsequently, participants in the Wake group were instructed not to nap. Participants in the Sleep group were equipped with polysomngraphy electrodes in their home, 1 h prior to their typical bedtime. Twelve hours after session 1, session 2 took place in the lab. Subjects completed the SSS followed by the Delayed Recall phase of the word-pair task.

### Questionnaires

During Session 1, participants completed an in-house questionnaire that recorded self-reported number of TBIs, cause of TBI(s), presence of post-concussive syndrome, and symptoms present after the injury. To assess the participant’s sleepiness level, or tendency to fall asleep during certain everyday activities, the ESS was completed (Johns, 1994). The PSQI was administered to assess the participant’s habitual sleep quality and sleep disturbances during the past month of sleep (Buysse et al., 1989). The MEQ was used to determine the participant’s chronotype, or preference for performing tasks and activities during the morning hours or evening hours (Horne and Ostberg, 1976). The SSS was used to determine the participant’s level of sleepiness at the time of the session (Hoddes et al., 1973). Lastly, the participants in the Sleep group were given a sleep survey to gather subjective information about the participant’s sleep during the night prior.

We used the Forward Digit Span task (FDS) from the Wechsler Adult Intelligence Scale IV to assess possible working memory deficits in the TBI group (Dean and Sterr, 2013). The FDS requires a series of digits to be read to the subject, who is then asked to verbally repeat the digits in the same order. This task begins with a series of 2 digits and increases in length by a single digit per trial up to a series of 10 digits (Wechsler, 2008). Due to late adoption of this measure, the FDS was performed by 18 participants in the TBI group and 24 in the non-TBI group.

### Polysomnography

Polysomnography was recorded with the Aura PSG ambulatory system (Grass Technologies). An electrode montage was applied in the participants’ home approximately 1 h before their typical bedtime. The montage included 2 EOG leads (right and left ocular canthus), two chin EMG leads, and six cortical EEG leads (F3, F4, C3, C4, O1, O2) with each electrode referenced to Cz. Data analysis was conducted according to the revised AASM manual (Silber et al., 2007).

## Data Analysis

### Word-Pair Learning and Consolidation

Word pair data were individually reviewed for total number of correct word pair responses, allowing for misspelling. Recall accuracy was measured as percentage of the total number of word pairs (of 36 possible). The change in performance between Immediate and Delayed Recall was assessed using an Intersession Change score. Intersession Change in recall was calculated by subtracting the Immediate Recall accuracy from the Delayed Recall accuracy and normalizing to baseline accuracy.

Intersession Change was compared across groups using *t*-tests to determine whether there was a difference between Sleep and Wake group performance. *T*-tests were also used to compare sleep staging and spectral power between TBI Sleep and non-TBI Sleep groups. Circadian effects at time of encoding were tested using a 2 × 2 ANOVA, with between-subject variables condition (Sleep vs. Wake) and group (TBI vs. non-TBI). Similarly, ANOVA tests were used to compare subjective sleep scores (SSS, ESS, PSQI, MEQ), cognitive performance on the FDS, and Intersession Change. Finally, Pearson’s correlations were performed to investigate relationships between sleep factors (e.g., WASO and %SWS) with Intersession Change to determine whether specific qualities of sleep are related to memory performance. Given the large number of statistical analyses, alpha was set to 0.01.

### Polysomnography

EEG, EMG, and EOG data were scored using the American Academy of Sleep Medicine (AASM) Manual for the Scoring of Sleep and Associated Events (Silber et al., 2007), identifying periods of wake and sleep stages NREM1, NREM2, SWS, and REM. Two trained sleep researchers scored all polysomnograms. Inconsistencies were discussed between scorers until consensus was reached. Total sleep across the night and percent in each sleep stage was calculated and compared across TBI and non-TBI groups. A combined %NREM2 and %SWS composite (%NREM) was created to address sleep stage interaction effects (Clemens et al., 2005; Göder et al., 2007). Pearson’s *R* coefficients were used to assess correlations between percent total sleep time spent in each sleep stage and intersession change in recall.

The spectral power density (μV^2^/Hz) in the sigma (12–15 Hz) and delta (0.5–4 Hz) range was quantified using BrainAnalyzer 2.0 Software (BrainVision, Berlin, Germany). Delta was examined during SWS, and sigma was examined in both NREM2 and over a combined NREM2 and SWS measure (Clemens et al., 2005; Holz et al., 2012). EEG data was first segmented into stages and filtered to 0.3–25 Hz. Raw data inspection was performed to eliminate artifacts, and data were again segmented into 4 s sections. Semi-automatic artifact rejection was performed on individual channels. Fast-Fourier transformation was performed with a Hanning window with 10% overlap. Analyses with delta and sigma utilize relative power (Khoury et al., 2013) in which power in the given spectrum was divided by time in the sleep stage measured (i.e., delta power divided by minutes in SWS; sigma power divided by minutes in NREM2 and NREM2/SWS combined).

## Results

### Group Descriptions

One TBI Sleep participant, 1 TBI Wake participant, and 1 non-TBI Wake participant were excluded for falling above or below 3 standard deviations of the mean in terms of Intersession Change in recall. The final sample included 56 participants across the four groups (Table 1). We confirmed all Wake participants slept >5 h the night before the experiment and all Sleep participants slept >5 h the night of the experiment. No Wake participants reported napping during the 12-h interval between session 1 and 2. No participants reported using alcohol or sleep medications during the study. There were no significant differences in age, gender ratio, or years of education between groups (Table 1).

**Table 1:** **Participant demographics and sleep questionnaire**.

	TBI sleep	TBI wake	Non-TBI sleep	Non-TBI wake	TBI vs. Non-TBI *p*-value	Sleep vs. Wake *p*-value
*n*	14	12	15	15
Age (years)	20.4 ± 1.5	20.2 ± 1.5	19.8 ± 1.4	19.5 ± 1.3	0.11	0.41
Education (years)	14.8 ± 1.5	15.1 ± 1.2	14.5 ± 1.3	14.1 ± 1.1	0.10	0.29
PSQI	5.0 ± 3.1	6.2 ± 3.2	4.7 ± 2.7	4.4 ± 1.7	0.18	0.39
MEQ	51.8 ± 7.5	43.5 ± 8.9	46.4 ± 7.5	45.7 ± 9.5	0.42	0.08^†^
SSS IR	3.1 ± 1.3	2.2 ± 0.6	2.9 ± 1.4	3.1 ± 1.2	0.34	0.18
SSS DR	2.4 ± 1.3	2.6 ± 1.7	2.7 ± 1.2	2.4 ± 1.2	0.89	0.96
ESS	7.5 ± 2.7	7.6 ± 3.8	9.3 ± 3.5	6.5 ± 3.4	0.68	0.18
Digit span	10.5 ± 1.9	11.4 ± 1.6	10.8 ± 1.7	12.2 ± 2.1	0.27	0.15

*Comparisons between TBI and non-TBI groups and also between Sleep and Wake groups. Mean ± standard deviation of participant demographics and questionnaire results. PSQI, Pittsburgh Sleep Quality Index; MEQ, Morning-Evening Questionnaire; SSS, Stanford Sleepiness Scores; IR, Immediate Recall; DR, Delayed Recall; ESS, Epworth Sleepiness Scale. ^†^ = p ≤ 0.08*.

There were no significant differences between TBI Sleep and Wake groups for TBI history or symptoms. Among the 26 TBI participants, there were 34 diagnosed concussions (19 in the Sleep group and 15 in the Wake group; six participants had more than one diagnosed TBI), 11 of which were accompanied by post-concussive syndrome. The average time since most recent concussion was 4.35 ± 3.14 years. The most common symptoms in both groups were dizziness, headache, decreased ability to concentrate, and fatigue (Table 2). Interestingly, only 2 TBI Wake participants and no TBI Sleep participants reported (TBI questionnaire) having sleep disturbances since the time of TBI.

**Table 2:** **Self-reported post-concussion symptoms**.

*“Following TBI, which did you experience?”*	Sleep	Wake
Post concussive syndrome (#)	8	3
Loss of consciousness (%)	35.7	41.7
Amnesia (%)	21.4	16.7
Nausea (%)	42.9	58.3
Vomiting (%)	21.4	16.7
Dizziness (%)	100	83.3
Headache (%)	92.9	75
Fatigue (%)	64.3	41.7
Decreased ability to concentrate (%)	64.3	83.3

Groups also did not differ in terms of habitual sleep or sleepiness or chronotype. As shown in Table 1, no significant differences were observed in PSQI, SSS at Immediate Recall, SSS at Delayed Recall, ESS, or MEQ scores. Importantly, no significant differences were found for FDS scores, indicating that TBI condition did not affect short-term memory and attention.

### Word Pair Learning Task

There were no significant differences in baseline performance across all groups as measured by Immediate Recall accuracy, *F*_(3,52)_ = 0.655; *p* = 0.58, or by number of rounds to reach criteria, *F*_(3,52)_ = 1.51, *p* = 0.22, indicating circadian effects and time-of-day did not affect performance (Table 3). Moreover, there was no significant difference in Immediate Recall for TBI and non-TBI groups, *t*_(52)_ = −1.38, *p* = 0.187, or for the number of rounds to reach criteria, *t*_(52)_ = 0.345, *p* = 0.63, suggesting that the history of TBI did not impair word-pair learning.

**Table 3:** **Word-pair task performance**.

	TBI Sleep	TBI Wake	non-TBI Sleep	non-TBI Wake	TBI vs. Non-TBI *p*-value
*n*	14	12	15	15
Immediate (%)	73.6 ± 16.7	78.5 ± 15.1	75.5 ± 17.2	81.7 ± 17.2	0.93
Delayed (%)	73.0 ± 17.0	73.5 ± 15.5	74.0 ± 17.8	76.9 ± 16.7	0.63
Rounds (#)	3.5 ± 1.4	3.1 ± 1.4	3.6 ± 1.3	2.8 ± 1.7	0.73

*Mean ± standard deviation of immediate recall accuracy, delayed recall accuracy, and number of rounds to reach criteria. p-value = t-test analyses of variance between TBI Sleep and non-TBI Sleep groups*.

**Figure 1:**
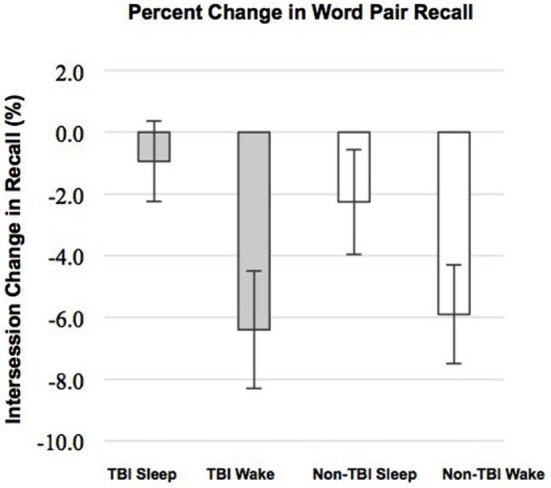
**Differences in Intersession Change between Sleep and Wake groups**. Error bars represent standard error of the mean.

We examined whether performance improvements were greater following sleep compared to wake, as predicted by our previous work (Wilson et al., 2012). There was a significant main effect of condition (Sleep vs. Wake) with greater reduction in recall in the Wake groups, *F*_(1,51)_ = 9.30; *p* = 0.004 (Figure 1). The main effect of group (TBI vs. non-TBI) was not significant, *F*_(2,51)_ = 0.421; *p* = 0.658, and there was no interaction between the Sleep/Wake condition and TBI/non-TBI status, *F*_(1,51)_ = 0.006; *p* = 0.939. These results indicate both Sleep groups significantly outperformed the Wake groups, and there were no differences in sleep-dependent consolidation between the TBI and non-TBI groups.

### Sleep Assessments

Due to equipment malfunction and operation errors, five sleep reports were unusable. The following results are based on polysomnography recorded in 11 participants in the TBI Sleep and 13 participants in the non-TBI Sleep group.

**Figure 2:**
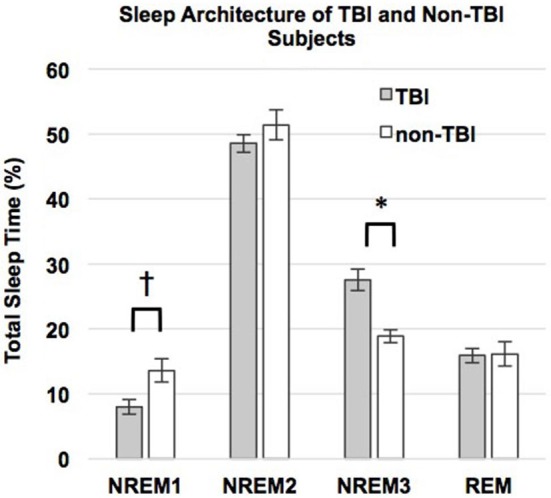
**Percent total sleep time spent in each stage of sleep**. Error bars represent standard error of the mean. ^†^ = *p* ≤ 0.08.

Contrary to our predictions, as shown in Table 4, there were no differences in sleep efficiency or WASO between the TBI and non-TBI Sleep groups. However, a two-tailed *t*-test showed a significant difference in %SWS between groups, where the TBI Sleep group had greater %SWS than the non-TBI Sleep group, *t*_(22)_ = 4.675, *p* = < 0.001, Table 4. This excess of SWS was at the expense of %NREM1, as an additional *t*-test revealed TBI participants had significantly less %NREM1 compared to non-TBI Sleep participants, *t*_(22)_ = −2.502, *p* = 0.02 (Figure 2), yet not when correcting for multiple comparisons. There were no differences between groups in total sleep time, sleep latency, %NREM2 or %REM. The TBI Sleep group spent more time, albeit not significantly more, in %NREM than did the non-TBI Sleep group, *t*_(22)_ = 1.96, *p* = 0.06, likely as a result of increased %SWS.

**Table 4:** **Sleep parameters**.

	TBI	non-TBI	*t*	*p*-value
Total sleep time (min.)	409.36 ± 50.28	395.54 ± 47.95	−0.77	0.49
Sleep latency (min.)	12.81 ± 8.30	23.19 ± 22.00	−1.47	0.15
WASO (min.)	20.72 ± 11.12	22.50 ± 11.12	−4.16	0.68
Sleep efficiency (%)	92.46 ± 2.10	89.68 ± 5.79	−1.51	0.15
%NREM1	8.01 ± 3.78	13.62 ± 6.55	−2.50	0.02^†^
%NREM2	48.53 ± 4.58	51.39 ± 8.29	−1.02	0.32
%SWS	27.56 ± 5.49	18.87 ± 3.55	−4.68	0.001*
%REM	15.89 ± 3.75	16.14 ± 6.67	−0.11	0.91
%NREM	76.48 ± 3.98	71.29 ± 8.20	−1.96	0.06^†^
Frontal Δ (μV2/Hz)	3.53 ± 1.51	4.64 ± 1.98	−1.35	0.20
Central Δ (μV2/Hz)	2.82 ± 1.31	3.80 ± 1.37	−1.78	0.08^†^
Frontal Σ (μV2/Hz)	0.016 ± 0.006	0.015 ± 0.008	−0.41	0.69
Central Σ (μV2/Hz)	0.016 ± 0.008	0.013 ± 0.007	−0.72	0.48
Frontal Σ (2&3) (μV2/Hz)	0.012 ± 0.004	0.012 ± 0.008	−0.023	0.98
Central Σ (2&3) (μV2/Hz)	0.010 ± 0.004	0.011 ± 0.006	−0.69	0.49

Mean ± standard deviation of sleep measurements obtained via polysomnography. WASO = wake after sleep onset; Δ = relative spectral power in the delta range during SWS; Σ = relative spectral power in the sigma range during NREM2; Σ (2&3) = relative spectral power in the sigma range over both NREM2 and SWS; p-value = t-test analyses of variance between TBI Sleep and non-TBI Sleep groups. *indicates significant. ^†^ = p ≤ 0.08.

Spectral power in the relative sigma (12–15 Hz) and relative delta (0.5–4 Hz) bands was compared between TBI and non-TBI Sleep groups using two-tailed *t*-tests. No differences were found in sigma power, or a combined NREM2/SWS sigma measure at any frontal or central locations. However, the central delta band differed near-significantly between the groups, *t*_(22)_ = −1.78, *p* = 0.08, such that that TBI group had lower relative delta power, as has been previously found (Khoury et al., 2013).

### Sleep and Memory Performance

**Figure 3:**
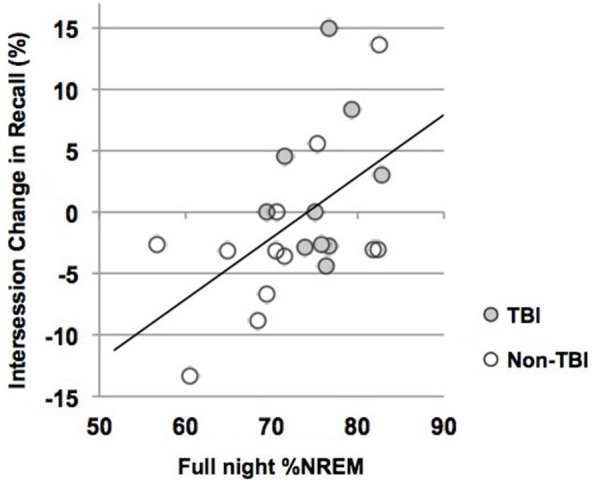
**Intersession Change and percent of the night in NREM (%NREM2 + %SWS)**. Line represents line of best fit for combined groups.

We used correlations to assess the relationship between sleep parameters and Intersession Change, a measure of memory consolidation. Given that there were no differences in the Intersession Change between the TBI and non-TBI groups, we initially considered these groups combined, and then analyzed the both the non-TBI and TBI groups separately. Counter to previous work (Holz et al., 2012), there was no significant correlation between Intersession Change and %SWS, %NREM2, delta power, sigma power in NREM2 or sigma power combined over NREM2 and SWS in any of the groups (Table 5). There was, however, a significant positive correlation between total %NREM and Intersession Change in the combined group (see Figure 3, *r* = 0.516; *p* = 0.01) and a near-significant positive correlation with Intersession Change (*r* = 0.56; *p* = 0.05) in the non-TBI group.

**Table 5:** **Intersession change and sleep parameter correlations**.

	TBI		non-TBI		Combined group	
	*r*	*p*-value	*r*	*p*-value	*r*	*p*-value
%NREM2	−0.09	0.78	−0.39	0.21	−0.19	0.37
%SWS	−0.13	0.69	−0.42	0.18	−0.32	0.13
%NREM	−0.27	0.40	−0.56	0.05^†^	−0.52	0.01*
Frontal Δ (μV2/Hz)	−0.24	0.53	−0.24	0.60	−0.26	0.33
Central Δ (μV2/Hz)	−0.24	0.54	−0.29	0.37	−0.32	0.17
Frontal Σ (μV2/Hz)	−0.64	0.09	0.003	0.99	−0.25	0.35
Central Σ (μV2/Hz)	−0.05	0.90	−0.04	0.90	−0.02	0.93
Frontal Σ (2&3) (μV2/Hz)	−0.63	0.09	−0.18	0.68	−0.32	0.23
Central Σ (2&3) (μV2/Hz)	−0.15	0.68	−0.09	0.77	−0.13	0.57

Correlation coefficient and p-value statistics for Pearson’s correlations between Intersession Change scores and sleep parameters. Δ = relative spectral power in the delta range during SWS; Σ = relative spectral power in the sigma range during NREM2; Σ (2&3) = relative spectral power in the sigma range over both NREM2 and SWS; p-value = t-test analyses of variance between TBI Sleep and non-TBI Sleep groups. *indicates significant. ^†^ = *p* ≤ 0.08.

Given that NREM during the first half of the night is particularly beneficial for memory consolidation (Plihal and Born, 1997) we performed a *post hoc* correlation between %NREM and Intersession Change in each half of the night, including all participants (values not reported). Consistent with prior reports, %NREM in the first half of the night was nearly positively correlated with Intersession Change (*r* = 0.437; *p* = 0.04) but this relationship was not present for half two (*r* = 0.228; *p* = 0.19).

## Discussion

This study investigated whether young adults with a history of TBI have decreased sleep quality and architecture and whether this affects sleep-dependent declarative memory consolidation. Contrary to our predictions, TBI subjects did not have increased WASO or decreased sleep efficiency. However, we report an increase in %SWS sleep, at the expense of %NREM1 sleep, in individuals who have experienced a TBI. This study is also the first to show preserved declarative sleep-dependent memory consolidation in those with a history of TBI. Interestingly, sleep-dependent memory consolidation was exhibited despite atypical sleep architecture.

As has been previously reported, after performing a declarative word-pair learning task, those who slept had a significantly greater Intersession Change than those who remained awake for an equivalent period of time, a hallmark of sleep-dependent memory consolidation. We do not believe this difference in recall is due to circadian effects, as there were no differences between learning at Immediate Recall for those who learned in the morning (Wake groups) and those who learned in the evening (Sleep groups).

Moreover, a significant correlation between %NREM and Intersession Change support an active role of sleep in memory consolidation (Holz et al., 2012; Wilson et al., 2012). We did not find a correlation between %SWS, %NREM2, or spindles and Intersession Change as reported in previous studies (Clemens et al., 2005; Genzel et al., 2009; Holz et al., 2012; Ruch et al., 2012). However, the association between Intersession Change and %NREM is consistent with hypotheses that sleep stages may interact or act sequentially (Mazzoni et al., 1999; Ficca et al., 2000; Clemens et al., 2005; Göder et al., 2007; Ruch et al., 2012). For example, recent studies using experimental sleep stage manipulations have found enhancing both NREM2 and SWS boosts declarative sleep-dependent consolidation (Mednick et al., 2013), whereas enhancing only SWS does not (Feld et al., 2013). It has therefore been suggested that the shared components of NREM2 and SWS (e.g., sleep spindles, slow oscillations) underlie consolidation in concert (Ackermann and Rasch, 2014). Thus, it may be that both NREM2 and SWS are important for declarative sleep-dependent memory consolidation. Of note, given that a large-scale investigation recently found no correlation between sleep staging and sleep-dependent consolidation of declarative memory traces (Ackermann et al., 2014), the relationship between %NREM and memory enhancement in the current study should be interpreted with caution.

Despite prior studies showing that short-term memory is affected in those who have suffered from a TBI (Dean and Sterr, 2013), we did not see deficits in sleep-dependent memory consolidation for those with and without a history of TBI. It has been posited that SWS facilitates restoration following brain injury (Gao et al., 2010; Sommerauer et al., 2013), and it may be that the normal-to-increased amount of SWS, which is a stage that is beneficial for cortical communication and plasticity, conserved both short-term memory and sleep-dependent consolidation in this sample.

Previous studies found decreased sleep efficiency and increased WASO in those with TBI (Kaufman et al., 2001; Parcell et al., 2008; Shekleton et al., 2010), yet we did not find a difference in these measures between groups in the present study. Differing sample demographics may account for these discrepancies in findings. Kaufman et al. (2001) studied younger individuals (10–16.5 years), who have considerably different sleep characteristics and needs than our young adult sample. Participants in the young adult age range are often sleep deprived and may therefore have a higher homeostatic sleep drive than younger adults (Hershner and Chervin, 2014). An increased homeostatic drive may minimize awakenings, which ultimately increases sleep efficiency. Additionally, Parcell et al. (2008) investigated individuals with a history of moderate to severe brain injury, whereas our sample had only mild TBI. It may be that poorer sleep quality accompanies more severe brain injury, and that sleep efficiency remains intact following a mild brain injury in young adults. Finally, because none of the TBI Sleep individuals reported sleep issues following injury, it may be that, by chance, our sample did not include subjects with poor sleep quality.

An increased %SWS was previously found in those with a history of TBI (Parcell et al., 2008; Shekleton et al., 2010; Sommerauer et al., 2013). Consistent with these findings, our non-TBI sample had %SWS in the normal range (~20%), and the TBI group had an unusually high %SWS compared to previous reports (Ohayon et al., 2004), with some exceeding 30%. Additionally, the TBI group had notably lower normalized delta power during SWS. There are multiple hypotheses that attempt to explain increased SWS post-TBI. First, following a series of investigations that found increased sleep need after both acute and chronic TBI, it was posited that SWS and its neuroplastic processes (e.g., axonal sprouting, synaptic remodeling) act as a recovery mechanism that aid in healing (Baumann et al., 2007; Kempf et al., 2010; Sommerauer et al., 2013). This hypothesis is supported by investigations in which SWS accelerates healing following a stroke (Gao et al., 2010). Paralleling this hypothesis, Parcell et al. (2008) suggested TBI may trigger a compensatory mechanism that occurs in response to brain injury, and this mechanism may increase %SWS. This hypothesis is supported by the increased neuronal reorganization, synaptic potentiation and neural proliferation seen following brain injury (Kernie et al., 2001; Nudo, 2011). Strengthening of neurons in response to injury may contribute to the synchronicity required for initiation and maintenance of %SWS. Finally, it has been suggested that the trauma itself increases SWS, as most brain injuries have a widespread affect on multiple brain circuits (Sommerauer et al., 2013). Given the current results, we posit that increased %NREM may be necessary to compensate for marginally lower delta power in the central sites.

At the expense of increased %SWS, the TBI individuals exhibited decreased %NREM1. Of note, %NREM1 found in the current sample is higher than that reported previously in young adults (15–20% vs. 2–5%: Ohayon et al., 2004). However, a closer examination of previous work shows that whereas %NREM1 is low when nocturnal sleep follows an adaptation night (Martin et al., 1997; Fischer et al., 2002), nights without an adaptation period often have NREM1 in a range of 14–23% (Crowley et al., 2002; Baran et al., 2012).

It is important to address the potential limitations of this study. Our sample included subjects who suffered from a concussion at least 1 year prior to enrollment, as we sought to include only those with long-term consequences of TBI. However, we did not require neurological records to verify TBI presence and severity. It is therefore possible that the TBI group included participants who did not suffer from a concussion as reported. Likewise, the non-TBI group may have included individuals who have indeed suffered from a concussion. However, given that we provided equal compensation for participants in both groups, it is doubtful that participants would deliberately falsely report their TBI history.

This sample lacked sleep efficiency and WASO deficits, as have been shown previously. They also lacked cognitive impairment, indicated by Digit Span scores and also unimpaired Immediate Recall performance. It may be, then, that our sample included an atypical population that is not representative of others with acute TBI. The heterogeneity of our sample (e.g., time since TBI, site of injury, etc.) may account for a lack of overlap with previous reports. However, given that differences observed in %SWS and relative delta power are consistent with prior work (Parcell et al., 2008; Shekleton et al., 2010; Khoury et al., 2013), it is unlikely that our sample was dissimilar from others. These results, in fact, highlight the severity of mild TBI, as our heterogeneous group in the chronic TBI stage exhibits characteristics that have been found in more homogenous groups with acute injury. Nonetheless, future work would benefit from neurological exams characterizing the TBI and identifying a more uniform group.

## Author Contributions

JM collected data, performed data analysis, wrote the manuscript; KMM collected data, performed data analysis; OSH collected data, performed data analysis; RMCS designed the study, wrote the manuscript.

## Conflict of Interest Statement

The authors declare that the research was conducted in the absence of any commercial or financial relationships that could be construed as a potential conflict of interest.
